# Biphasic activation of complement and fibrinolysis during the human nasal allergic response

**DOI:** 10.1016/j.jaci.2018.01.022

**Published:** 2018-05

**Authors:** Ryan S. Thwaites, Natasha C. Gunawardana, Verena Broich, Elizabeth H. Mann, Josefin Ahnström, Gaynor A. Campbell, Sarah Lindsley, Nehmat Singh, Tanushree Tunstall, David A. Lane, Peter J. Openshaw, Catherine M. Hawrylowicz, Trevor T. Hansel

**Affiliations:** a National Heart and Lung Institute, Faculty of Medicine, Imperial College London, London, United Kingdom; b MRC and Asthma UK Centre for Allergic Mechanisms of Asthma, King's College London, Guy's Hospital, London, United Kingdom; c Centre for Haematology, Faculty of Medicine, Imperial College London, London, United Kingdom; d MRC and Asthma UK Centre, Imperial College London, London, United Kingdom

To the Editor:

The interlinked pathways of complement activation, coagulation, fibrinolysis, and fibrosis contribute to numerous respiratory diseases.^1^, ^2^, ^3^, ^4^ Multiple proteases, including mast cell tryptase released in the allergic reaction, have been documented to activate complement.^5^ In an extensive gene expression analysis, we recently observed changes in complement factors in mucosal curettage samples following nasal allergen challenge (NAC).^6^ Although complement activation has been reported in response to allergen,^7^ it has hitherto not been characterized as a detailed *in vivo* kinetic response. We therefore investigated the kinetics of allergen-induced activation of the complement and coagulation cascades in nasal mucosal lining fluid.

The NAC protocol, demographic characteristics, and clinical response to challenge are summarized in the Results section and Fig E1 in this article's Online Repository at www.jacionline.org. Two phases of the allergic response have been described: the early allergic reaction (EAR, between 0 and 2 hours post-NAC) and the late allergic reaction (LAR, >2 hours post-NAC).^6^ Levels of prostaglandin-D2 (PGD2) rose rapidly, peaking at 5 minutes in the EAR and returned to baseline by 1 hour post-NAC (Fig 1, *A*), probably resulting from mast cell activation; these kinetics closely match a previous observation of β-tryptase release by mast cells.^8^ Levels of the activated complement components C3a, C4a, and C5a peaked at 30 to 45 minutes, with a second peak observed at 480 minutes (Fig 1, *A*). All data in Fig 1 are presented in full in Fig E2 in this article's Online Repository at www.jacionline.org. The 30-45 minute peak of complement activation was universal among volunteers, where such early peaks likely represent activation of complement preexistent in the airway. In contrast, the second peak was clearly evident only in some volunteers. Matrix metalloprotease 9 (MMP9) and the type II mediators IL-5 and IL-9 were significantly elevated in the LAR, peaking at 480 minutes (Fig 1, *B*).

**Fig 1:**
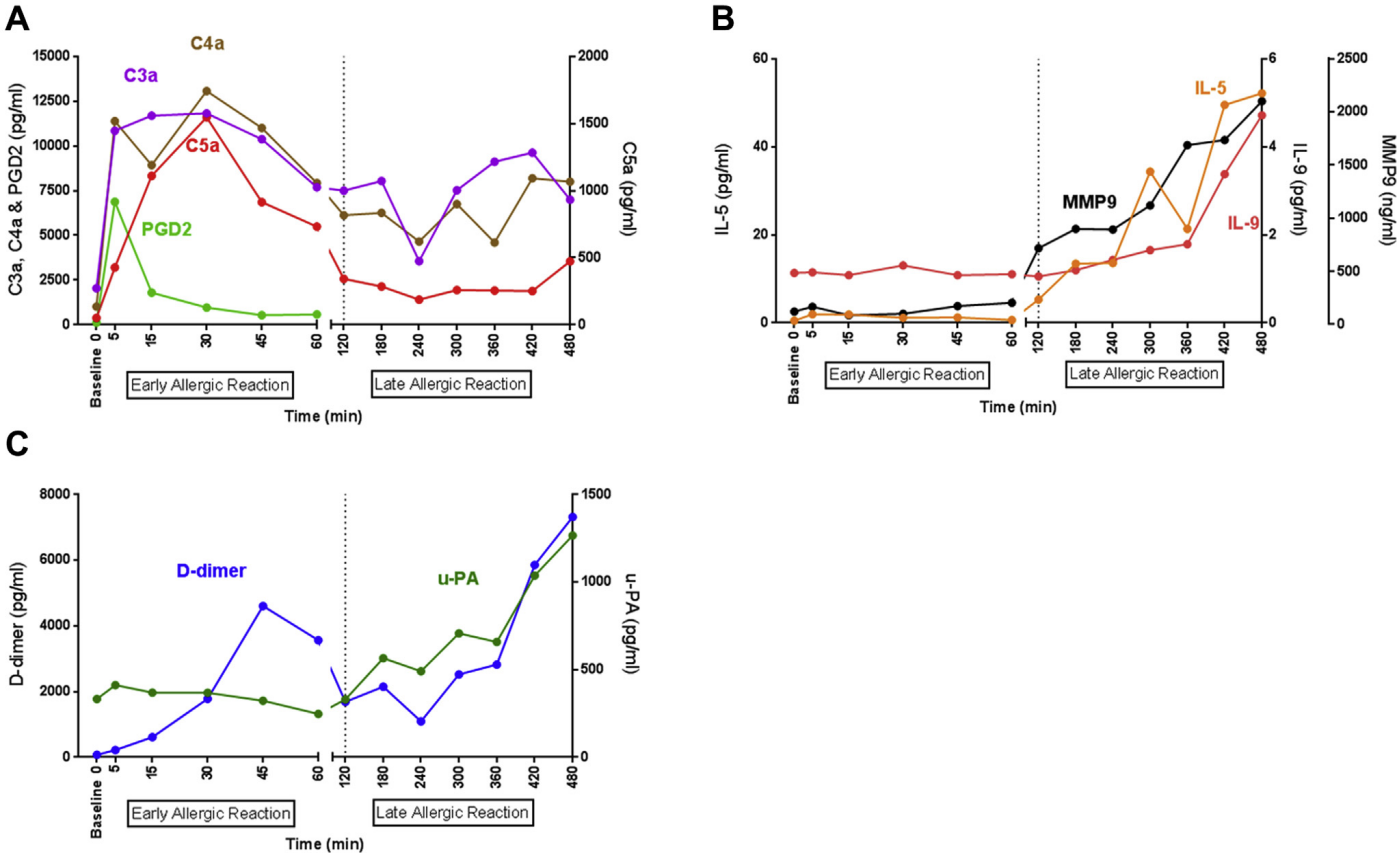
Mast cell degranulation, type II inflammation, complement activation, and fibrinolysis following NAC. Following NAC, nasosorption was used to measure the levels of inflammatory mediators: **(A)** PGD2 in the first hour and the active complement components C3a, C4a, and C5a over the 8-hour time series. In addition, levels of **(B)** IL-5, IL-9, and MMP9 are shown over 8 hours. **C,** D-dimer and u-PA levels post-NAC. Data are represented as medians (n = 15). See Fig E2 for statistical analyses. *MMP9*, Matrix metalloprotease 9.

Because complement can activate the coagulation cascade, we measured mucosal levels of coagulation factors. We observed high levels of tissue factor (TF) using a TF-dependent thrombin generation assay, but levels were unaltered by NAC (see Fig E3, *A*, in this article's Online Repository at www.jacionline.org). These data suggested that coagulation can occur in the upper airway without the need for local TF production post-NAC. Levels of intermediates of coagulation were below detection limits (see the Results section).

Coagulation generates fibrin that is cleared by fibrinolysis, resulting in the formation of fibrin degradation products including D-dimer. Strikingly, D-dimer levels also increased in a biphasic manner, reminiscent of complement components, with peaks observed at 45 and 480 minutes after NAC (Fig 1, *C*). Similarly to complement, late-phase D-dimer elevation was evident in only approximately 50% of participants. Fibrinolysis is plasmin-dependent and the plasminogen activation cascade has been associated with asthma and allergies,^1^, ^2^, ^3^ but no significant increases in tissue-type plasminogen activator nor plasminogen activator inhibitor 1 activity were observed (data not shown). In contrast, levels of urokinase-type plasminogen activator (u-PA) increased during the LAR (Fig 1, *C*; see Fig E2, *I*, in this article's Online Repository at www.jacionline.org).

A correlation matrix was established to determine the interrelation between markers of mast cell activation, complement activation, type II inflammation, and fibrinolysis during the EAR and LAR. The matrix analyzed area under the curve values of the induction of mediators over the EAR and LAR (Fig 2, *A*). The *P* values and Spearman *R* scores of this matrix are listed in Tables E1 and E2, respectively, in this article's Online Repository at www.jacionline.org.

**Fig 2:**
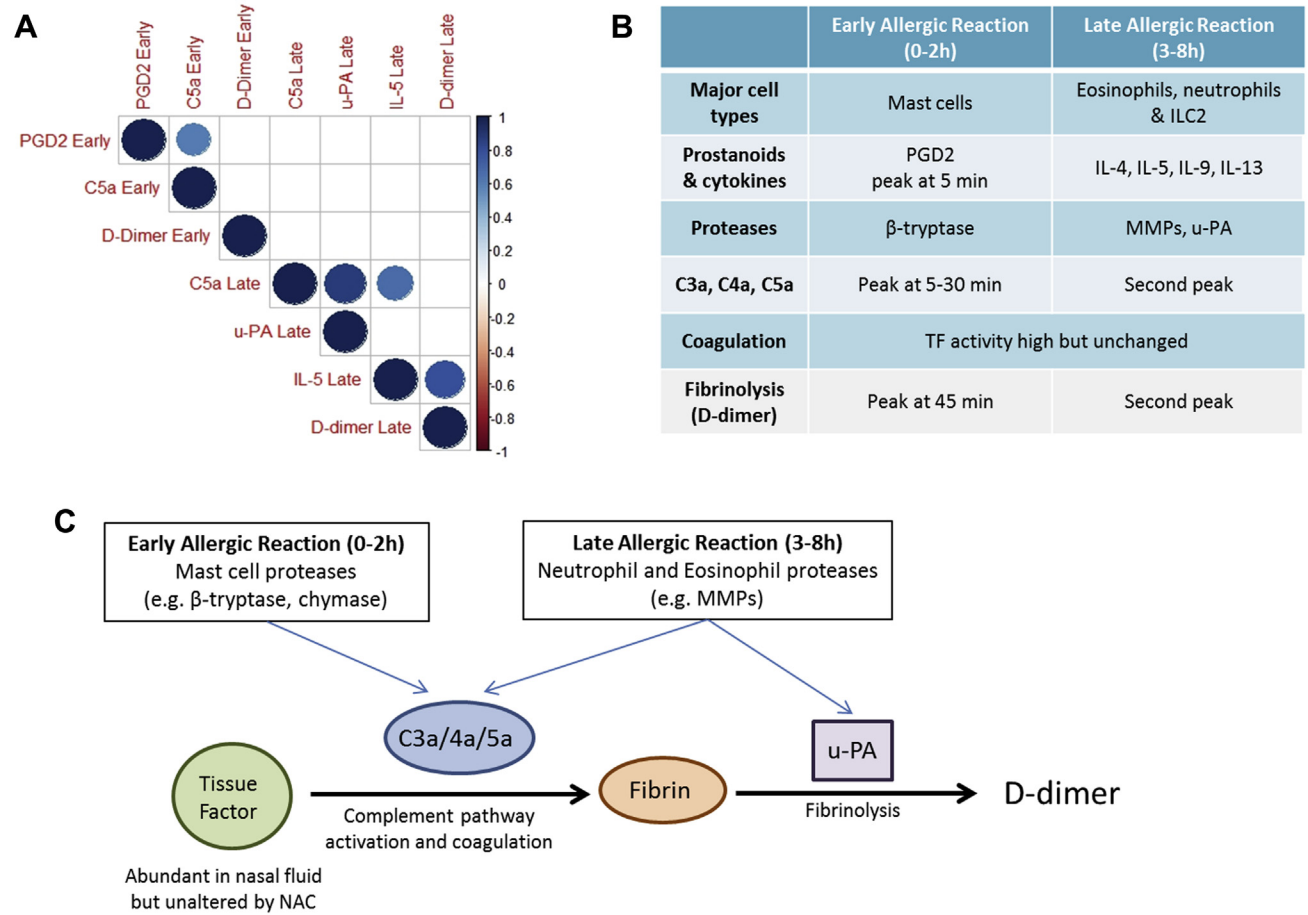
Distinct phases of complement activation and fibrinolysis are associated with the EARs and LARs. **A,** Correlation matrix of the induction of each mediator over the early (0-2 hours) and late (3-8 hours) allergic reactions; blank squares denote insignificant (*P* > .05) correlations, and color denotes Spearman *R* value. **B,** Activation of the complement and coagulation cascades in the EAR and LAR is summarized, along with cells and mediators likely to be implicated in these processes. **C,** Proposed model of the distinct phases of complement activation in the EARs and LARs. In the early phase, mast cell products trigger complement activation, which, in turn, activates TF, which is abundant in the upper airway, resulting in fibrin deposition. Fibrinolysis rapidly follows, resulting in D-dimer formation. In the late phase, complement may be activated by proteases associated with type II inflammation, similarly triggering fibrin deposition through TF activation. In the late phase, u-PA levels rise, resulting in increased plasmin generation from plasminogen. Plasmin contributes to fibrinolysis and D-dimer formation.

Interestingly, EAR PGD2 levels, a measure of mast cell activation, did not correlate with any LAR response, including type II inflammation. However, PGD2 and early C5a levels were significantly associated (*P* = .04; Fig 2, *A*). Trends were observed between C5a and D-dimer levels, in both the EAR and LAR (*P* = .12 and *P* = .07, respectively). LAR levels of C5a correlated with u-PA and IL-5 (*P* = .0009 and *P* = .007, respectively; Fig 2, *A*). In addition, a strong correlation was observed between IL-5 and D-dimer (*P* = .003, Fig 2, A). These results suggested that complement activation in the EAR was coupled with mast cell activation, whereas a second wave of complement activation and fibrinolysis in the LAR was associated with u-PA and type II inflammation.

This study details the kinetics of complement activation and fibrinolysis in the upper airway following NAC, extending a previous report of gene expression.^6^ Validation of nasosorption and biomarker assessment will be required in patients with allergic rhinitis in the context of natural seasonal exposure. This will establish whether these nasal mucosal biomarkers are useful in diagnosis, stratification, and monitoring in patients with allergic rhinitis. Early complement activation is in line with cleavage by mast cell tryptase,^5^ whereas in the LAR, associations between C5a, IL-5, u-PA, and D-dimer suggest that complement activation may be a component of type II inflammation (Fig 2, *B* and *C*), where proteases such as MMP9 could activate complement. In asthma, sputum eosinophils have been reported to correlate with D-dimer levels^2^ and complement anaphylatoxins have been associated with maturation of maladaptive immune responses. Similarly, extensive complement deposition in nasal polyps of patients with chronic rhinosinusitis is closely associated with local IgM and anti-DNA IgG levels.^9^ In addition, the complement and coagulation cascades contribute to idiopathic pulmonary fibrosis.^3^, ^4^ Activation of these pathways may represent a common feature of numerous airways diseases and contribute to components of remodeling and fibrosis. Therefore, analysis of complement, coagulation, and fibrinolysis cascades in airway mucosal lining fluids will be of interest in a range of respiratory diseases.

## Figures and Tables

**Fig E1:**
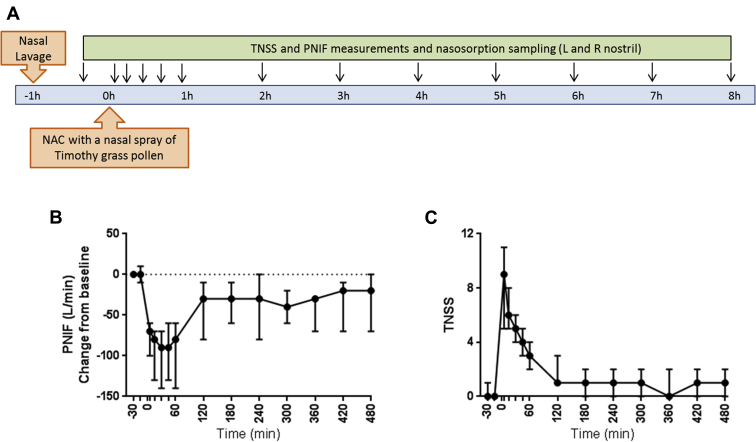
Study design and clinical response. **A,** Clinical events during NAC. Nasal lavage was performed before allergen administration. Nasosorption sampling and measurements for total nasal symptom score (TNSS) and peak nasal inspiratory flow (PNIF) were taken at 5, 15, 30, 45, and 60 minutes post-NAC, then hourly to 480 minutes. Clinical severity of the allergic reaction was measured by **(B)** PNIF and **(C)** TNSS at each time point. N = 15. *L*, Left; *R*, right. Data are represented as medians with interquartile ranges.

**Fig E2:**
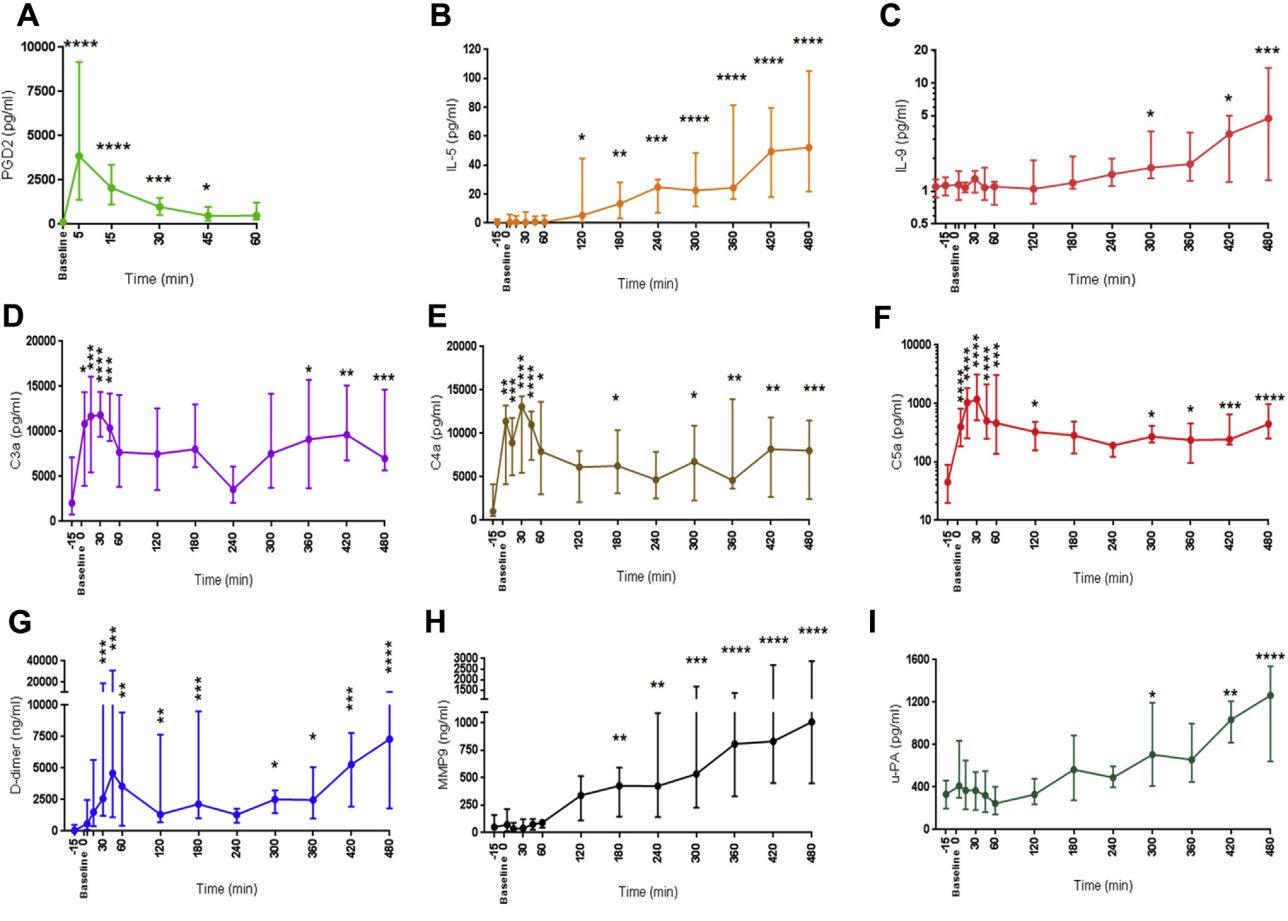
Mediator levels following NAC. Data from Fig 1 are shown as medians and interquartile ranges. Following NAC, mediators and markers of mast cell degranulation, type II inflammation, complement activation, fibrinolysis, and plasminogen activation were measured: **A,** PGD2 in the first hour post-NAC. **B,** IL-5. **C,** IL-9. The complement components (**D)** C3a, (**E)** C4a, and (**F)** C5a. In addition, levels of (**G**) D-dimer, (**H**) MMP9, and (**I**) urokinase (u-PA) were determined (n = 15). Friedman test with Dunn correction for multiple comparisons, based on the level at each time point relative to those at baseline, was used to determine statistical significance (*P < .05, **P < .01, ***P < .001, ****P < .0001). *MMP9*, Matrix metalloprotease 9.

**Fig E3:**
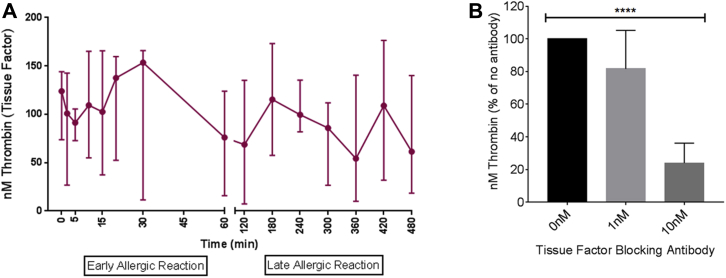
Tissue Factor (TF) is persistently present in the human airway at baseline and during allergen exposure. **A,** TF levels in the nose were indirectly determined using a TF-dependent thrombin generation assay, carried out at baseline and for 8 hours following NAC (n = 4). Data are represented as the median and interquartile range of the subjects, where the thrombin generation of each sample was determined in 3 independent technical replicates. **B,** To confirm that thrombin generation was TF dependent, thrombin generation was determined in the presence of an inhibitory anti-TF antibody (1 or 10 nM) (n = 8). The thrombin generation of these 8 samples (20 minutes pre- and post-NAC from 4 donors) was determined as the mean of 2 independent technical replicates. Data are shown as mean ± SEM of percentage change from the 0 nM control in each sample. Inhibition data were tested for significance by 1-way ANOVA (****P < .0001).

**Table E1:** Correlation matrix significance values

Mediator (reaction phase)	PGD2 early	C5a early	D-dimer early	C5a late	u-PA late	IL-5 late	D-dimer late
PGD2 early	.000	.037	.439	.964	.865	.600	.659
C5a early		.000	.087	.123	.295	.321	.247
D-dimer early			.000	.166	.407	.312	.508
C5a late				.000	.001	.007	.160
u-PA late					.000	.348	.051
IL-5 late						.000	.010
D-dimer late							.000

*P* values from the correlation matrix (Fig 2, *A*).

**Table E2:** Correlation matrix Spearman coefficients

Mediator (reaction phase)	PGD2 early	C5a early	D-dimer early	C5a late	u-PA late	IL-5 late	D-dimer late
PGD2 early	1.000	0.608	0.021	−0.057	−0.036	−0.082	0.070
C5a early		1.000	0.527	0.329	0.381	0.203	0.297
D-dimer early			1.000	0.427	0.238	0.287	0.175
C5a late				1.000	0.873	0.675	0.538
u-PA late					1.000	0.318	0.595
IL-5 late						1.000	0.797
D-dimer late							1.000

Spearman *R* values from the correlation matrix (Fig 2, *A*).

## Methods

### Measurement of total nasal symptom scores and peak nasal inspiratory flow

Total nasal symptom scores were scored by participants on a 12-point scale, with 0 to 3 points given to each of nasal congestion, itching, sneezing, and rhinorrhea (0 = none, 1 = mild, 2 = moderate, 3 = severe symptoms). Measurements were taken at each sampling time point. Nasal patency was measured by peak nasal inspiratory flow using a Youlten nasal peak flow meter with the highest result of 3 measurements used for analyses.

### Sampling

Nasal lavage was performed using 5 mL normal sterile saline on all volunteers 60 minutes before allergen administration. Nasal fluid was collected using nasosorption devices (Hunt Developments Ltd, Midhurst, UK). Sampling was performed at the inferior turbinate, with absorption performed for 60 seconds. Collections were performed at baseline (15 minutes before allergen administration), then at 5, 15, 30, 45, and 60 minutes postchallenge. After 60 minutes, samples were collected hourly up to 8 hours. After collection samples were returned to their storage tubes and immediately dry-frozen at −80°C until analysis. Fluid was eluted from the nasosorption matrix using 300 μL of Millipore assay buffer (Millipore, Billerica, Mass) and centrifugation at 16000*g* for 20 minutes, at 4°C. Samples for measurement of TF were eluted using the same protocol, with the exception that Tris-buffered saline replaced Millipore assay buffer.

### Mediator measurements

Eluted nasosorption fluid was analyzed by immunoassay for a range of inflammatory cascade components. Plasminogen activator inhibitor 1 was measured by ELISA using duoset antibodies as per manufacturer's instructions (R&D Systems, Minneapolis, Minn). PGD2 levels were measured using a PGD2-MOX ELISA kit (Cayman Chemicals, Ann Arbor, Mich). Activity of tissue-type plasminogen activator was measured using Combi Actibind ELISA kits (Technoclone, Vienna, Austria). Levels of the fibrin degradation product D-dimer were quantified using a HemosIL ACL Acustar D-dimer immunofluorescence assay (Instrumentation Laboratory, Bedford, Mass). A luminex assay was used to measure levels of urokinase (u-PA) (R&D Systems) using a Bioplex 200 analyzer (Bio-Rad, Hercules, Calif). C3a, C4a, and C5a levels were measured using the Luminex system with a cytometric bead array anaphylatoxin panel (BD Bioscience, San Jose, Calif). Levels of IL-5, IL-9, and matrix metalloprotease 9 were measured using MSD V-PLEX kits (MSD, Rockville, Md).

### Measurement of TF using calibrated automated thrombography

TF levels in nasosorption eluates were assessed indirectly in real-time thrombin generation assays by calibrated automated thrombography, using a Fluoroscan Ascent FL plate reader (Thermo Labsystems, Philadeliphia, Pa) and Thrombinoscope software (Synapse B.V., Maastricht, The Netherlands). Thrombin generation was initiated in 80 μL pooled normal plasma in the presence of 4 μM phospholipid vesicles and 16.6 mM CaCl_2_ in a total volume of 120 μL. The amount of thrombin formed was monitored using a thrombin-specific fluorogenic substrate, Z-Gly-Arg-AMC-HCl (0.42 mM; Bachem, Bubendorf, Switzerland). The nasosorption eluates were added in a 1:100 final dilution. To inhibit contact activation, corn trypsin inhibitor (Enzyme Research Laboratories, South Bend, Ind) was added to the plasma (40 μg/mL plasma). An inhibitory antibody against TF (1 or 10 nM; Sigma, St-Louis, Mo) was added to show that any thrombin generation detected in the eluted nasal fluids was caused by TF.

### Statistical analyses

Statistical analyses of time-course data were performed in GraphPad Prism v7.00 (GraphPad Software, La Jolla, Calif) using Friedman test with Dunn correction for multiple comparisons, based on the level at each time point relative to that at baseline. All plots are shown as medians with interquartile ranges. Correlation analyses were performed in R (R Foundation for Statistical Computing, Vienna, Austria) using the “ggcorrplot” package. All correlations were based on mediator data expressed as fold-induction over baseline of each individual. Statistical testing of these correlation plots used Spearman coefficients.

## Results

Fifteen adult volunteers sensitized to timothy grass pollen were recruited. The mean age of participants was 32.4 years (range, 19-57 years), and 8 participants were men. To enable measurements of TF levels, a second cohort (n = 4) was recruited, as detailed in the Methods section. In the main study, the average wheal diameter following skin prick testing with timothy grass pollen was 8 mm (range, 5-14 mm). The schedule for NAC and respiratory sampling, performed by nasosorption of mucosal lining fluid, is summarized in Fig E1, *A*. Following NAC, peak nasal inspiratory flow fell rapidly to a nadir in the EAR at 30 to 45 minutes (median, −90 L/min; Fig E1, *B*). Similarly, total nasal symptom score, which is scored out of 12, rose sharply, peaking at 5 minutes (median, 9; range, 3-11; Fig E1, *C*). In the LAR, clinical symptoms generally consisted of a mild nasal obstruction. The clinical magnitude of the EAR did not predict the clinical magnitude of the LAR. Furthermore, no association was observed between mediator levels and symptoms in the LAR (data not shown).

For measurement of TF, a second cohort of volunteers was challenged with timothy grass pollen using an identical challenge protocol. The mean age of these volunteers was 32 years (range, 27-39 years); 3 were men. The mean wheal diameter following skin prick testing with timothy grass pollen was 6.4 mm (range, 5.5-10 mm).

Levels of the coagulation cascade intermediates thrombin-antithrombin complex and prothrombin fragments 1 and 2 were measured in the main cohort (n = 15) but were below assay sensitivity (15.6 pg/mL and 0.007 nmol/mL, respectively; data not shown). In addition, no significant changes in plasminogen activator inhibitor 1 levels were observed, though levels were frequently below assay sensitivity (0.31 ng/mL; data not shown).
